# Tetrahydroquinazole-based secondary sulphonamides as carbonic anhydrase inhibitors: synthesis, biological evaluation against isoforms I, II, IV, and IX, and computational studies

**DOI:** 10.1080/14756366.2021.1956913

**Published:** 2021-08-02

**Authors:** Emma Baglini, Rahul Ravichandran, Emanuela Berrino, Silvia Salerno, Elisabetta Barresi, Anna Maria Marini, Monica Viviano, Sabrina Castellano, Federico Da Settimo, Claudiu T. Supuran, Sandro Cosconati, Sabrina Taliani

**Affiliations:** a Department of Pharmacy, University of Pisa, Pisa, Italy; b DiSTABiF, University of Campania Luigi Vanvitelli, Caserta, Italy; c NEUROFARBA Department, Sezione di Scienze Farmaceutiche e Nutraceutiche, Università degli Studi di Firenze, Sesto Fiorentino, Italy; d Department of Pharmacy, Epigenetic Med Chem Lab, University of Salerno, Fisciano, Italy

**Keywords:** Carbonic anhydrases inhibitors, secondary sulphonamides, tetrahydroquinazole derivatives, structure–activity relationships, tumour-related CA IX isoform

## Abstract

A library of variously decorated *N*-phenyl secondary sulphonamides featuring the bicyclic tetrahydroquinazole scaffold was synthesised and biologically evaluated for their inhibitory activity against human carbonic anhydrase (hCA) I, II, IV, and IX. Of note, several compounds were identified showing submicromolar potency and excellent selectivity for the tumour-related hCA IX isoform. Structure–activity relationship data attained for various substitutions were rationalised by molecular modelling studies in terms of both inhibitory activity and selectivity.

## Introduction

Carbonic anhydrases (CAs) are ubiquitous zinc enzymes that catalyse the reversible hydration of carbon dioxide to bicarbonate by a metal hydroxide nucleophilic mechanism^1^. CAs play key roles in several physiological processes, such as pH and CO_2_ homeostasis, respiration, secretion of electrolytes in a variety of tissues/organs, biosynthetic reactions, including lipogenesis, gluconeogenesis, ureagenesis, calcification, bone resorption, and tumorigenicity^2–5^. To date, 15 different isoforms of human CAs (hCAs) have been identified with different catalytic activities, cellular and subcellular localisation, and tissue distributions^6^: CA I, CA II, CA III, CA VII, and CA XIII isoforms are cytosolic, CA IV, CA IX, CA XII, CA XIV, and CA XV are transmembrane bound, CA VA and CA VB are mitochondrial and CA VI is secreted in saliva and milk^7^^,^^8^. Isoforms VIII, X, and XI are cytosolic, acatalytic, and preponderantly expressed within the brain, but their functions remain elusive.

**Chart 1.**
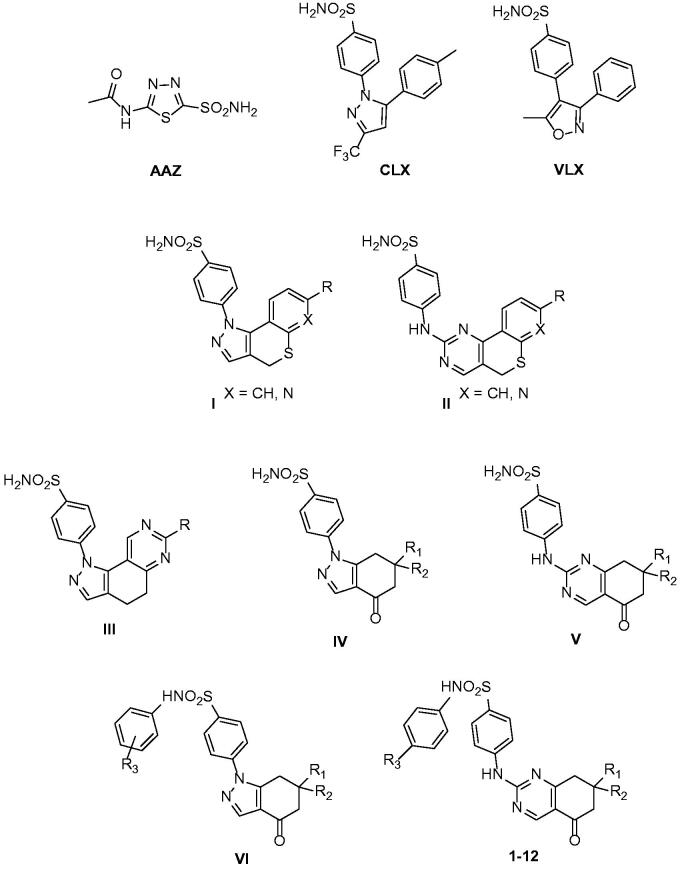
Structures of celecoxib (CLX), valdecoxib (VLX), acetazolamide (AAZ); structures of previously described (I-VI) and newly synthesized (1–12) CAIs.]

Many hCA isoenzymes are considered promising therapeutic targets in the development of potentially valuable new small molecules for the treatment or prevention of many diseases such as glaucoma, epilepsy, obesity, and cancer^9^^,^^10^. Indeed, CA inhibitors (CAIs) have been in clinical use for many decades as diuretics and systemically acting antiglaucoma drugs; many CAIs are primary sulphonamides (acetazolamide (**AAZ**, Chart 1), ethoxzolamide, dichlorphenamide) coordinating the zinc ion present in the catalytic site through their deprotonated sulphonamide nitrogen atom^11^.  However, the high sequence homology between hCA isoforms, combined with their widespread and ubiquitous tissue localisation, makes the discovery of CAIs with functional selectivity for a specific target isoform a major challenge in the development of potential drugs, free from off-target side effects^12^.

In the last decades, several tricyclic/bicyclic scaffolds, decorated with a primary sulphonamide moiety, have been exploited by our research group, leading to the identification of several derivatives with high CA inhibitory activity and promising selectivity profiles^13–15^. In particular, tricyclic primary benzenesulfonamides featuring the benzothiopyrano[4,3-*c*]pyrazole and pyridothiopyrano[4,3-*c*]pyrazole systems (see general formula **I**, Chart 1) were initially developed as geometrically constrained analogues of celecoxib (**CLX**, Chart 1) and valdecoxib (**VLX**, Chart 1), cyclooxygenase 2 (COX-2) specific inhibitors that act also as potent CAIs^6^^,^^13^. Based on the encouraging results obtained and the SAR outlined, our project on CAIs was pursued, always within primary benzenesulfonamide-based small molecules, following two different approaches: (i) investigation of three different, but structurally related, tricyclic scaffolds, that is benzo/pyrido-thiopyranopyrimidine **II** and pyrazolodihydroquinazoline **III** (Chart 1)^14^^,^^15^; (ii) structural simplification of the core scaffolds of **I** and **II** to yield bicyclic tetrahydroindazole **IV** and tetrahydroquinazole **V** derivatives, respectively, in which the phenyl- or pyrido-fused moiety was removed (Chart 1)^15^.

More recently, the tetrahydroindazole scaffold of class **IV** was further exploited by the development of a library of *N*-phenyl-secondary sulphonamides, variously decorated at para- or meta-position with groups characterised by different electron-donor/acceptor capabilities (**VI**, Chart 1)^16^. Indeed, although secondary and tertiary sulphonamides have been recently reported as efficient and selective inhibitors of the cancer-related hCA IX and XII isoforms,^17–25^ the chemical space of such CAIs remains so far rarely explored and their binding mode is still a subject of investigation^26^.

For what concerns class **VI**, although, in general, the insertion of a secondary sulphonamide function on the tetrahydroindazole scaffold produced inhibitors with a moderate micromolar potency, lower than the primary sulphonamide parent compounds of series **IV**, some interesting structure–activity relationship (SAR) data were acquired and rationalised through theoretical studies^16^. Specifically, the inhibitory profile of the novel indazolyl-*N*-arylbenzenesulfonamides **VI** appears strictly dependent on the substitution pattern at 6-position, with the 6-phenyl group able to produce hCA II inhibitors with good potency and selectivity^16^.

Pursuing our interest in the development of secondary sulphonamide CAIs, in the present study, we turned out our attention to the bicyclic tetrahydroquinazole scaffold of class **V**. Thus, derivatives **1**–**12** (Chart 1) were synthesised and biologically evaluated for their enzyme inhibitory activity against four physiologically relevant CA isoforms, the hCA I, II, IV, and IX. In the novel compounds, the secondary benzenesulfonamide moiety was decorated at *para* position with groups of various nature (R_3_=H, OCH_3_, NO_2_, CH_3_) to modulate the acidity of the sulphonamide itself, keeping in mind the groups conferring the best results, in terms of potency and selectivity, in series **VI**; in addition, the same substituents of the corresponding primary sulphonamides **V** described by us^15^ were maintained at 7-position (R_1_=R_2_=H; R_1_=H, R_2_=C_6_H_5_; R_1_=R_2_=CH_3_).

## Materials and methods

### Chemistry

The uncorrected melting points were determined using a Reichert Köfler hot-stage apparatus. NMR spectra were obtained on a Bruker AVANCE 400 (^1^H, 400 MHz, ^13^C, 100 MHz) in DMSO-d_6_. Chemical shifts are expressed in *δ* (ppm) and coupling constants (*J*) in Hertz. Magnesium sulphate was used as the drying agent. Evaporations were made *in vacuo* (rotating evaporator). Analytical TLC have been carried out on Merck 0.2 mm precoated silica gel aluminium sheets (60 F-254). Silica gel 60 (230–400 mesh) was used for column chromatography. Microwave (MW) assisted reactions were carried out in BIOTAGE Initiator 2.5 microwave apparatus. The purity of the target inhibitors **1**–**12** was determined, using a Shimadzu LC-20AD SP liquid chromatograph equipped with a DDA Detector (Kyoto, Japan) at 220 nm (column C18 (250 mm × 4.6 mm, 5 µm, Shim-pack)). The mobile phase, delivered at isocratic flow, consisted of methanol (80%) and water (20%) and a flow rate of 1.0 ml/min. All the compounds showed percent purity values of >95%. Reagents, starting materials, and solvents were purchased from commercial suppliers and used as received. The following intermediates were obtained according to previously described procedures: 2-[(dimethylamino)methylene]cyclohexan-1,3-dione **14a**^27^, 2-[(dimethylamino)methylene]-5-phenylcyclohexan-1,3-dione **14b**^27^, 2-[(dimethylamino)methylene]-5,5-dimethylcyclohexan-1,3-dione **14c**^27^, and *N*-(4-aminosulfonyl)phenylguanidine carbonate **15**^14^.

### General procedure for the synthesis of 4-((5-oxo-5,6,7,8-tetrahydroquinazolin-2-yl)amino)benzenesulfonamide derivatives 16a–c

A mixture of the proper 2-(dimethylamino)methylene-1,3-dione derivative **14a**–**c** (0.45 mmol), *N*-(4-aminosulfonyl)phenylguanidine carbonate **15** (0.25 g, 0.90 mmol) and NaOH (54 mg, 1.35 mmol) in 5 ml of *n*-BuOH was irradiated at a *T* = 160 °C (pre-stirring time = 3 min) for 40 min. After cooling, the obtained crude solid compounds **16a**–**c** were purified by recrystallisation from ethanol.

4-((5-oxo-5,6,7,8-tetrahydroquinazolin-2-yl)amino)benzenesulfonamide (**16a**). Yield 66%; m.p. 294–296 °C^15^.

4-((5-oxo-7-phenyl-5,6,7,8-tetrahydroquinazolin-2-yl)amino)benzenesulfonamide (**16b**). Yield 70%; m.p. 214–215 °C^15^.

4-((5-oxo-7,7-dimethyl-5,6,7,8-tetrahydroquinazolin-2-yl)amino)benzenesulfonamide (**16c**). Yield 72%; m.p. 258–260 °C^15^.

### General procedure for the synthesis of N-aryl-4-((5-oxo-5,6,7,8-tetrahydroquinazolin-2-yl)amino)benzenesulfonamide derivatives 1–12

To a solution of the proper 4-(5-oxo-7-substituted-5,6,7,8-tetrahydroquinazolin-2-yl)amino)benzenesulfonamide **16a**–**c** (0.94 mmol) in dry MeCN (10 ml) at room temperature, CuI (8 mg, 0.04 mmol), anhydrous K_2_CO_3_ (0.28 g, 2.07 mmol), the proper aryl iodide (1.13 mmol), and DMEDA (0.04 ml, 0.38 mmol) were added, and the resulting mixture was heated at 100 °C for 8–24 h (TLC analysis). After cooling, the reaction mixture was diluted with water (20 ml), acidified with 2.0 N HCl to pH = 4, and extracted with EtOAc (3 × 20 ml). The organic phase was dried (MgSO_4_), filtered and evaporated under reduced pressure. The crude material was purified by flash chromatography using PE/EtOAc as eluting system, to afford compounds **1**–**12**.

#### 4-((5-Oxo-5,6,7,8-tetrahydroquinazolin-2-yl)amino)-N-phenylbenzenesulfonamide (1)

Compound **1** was obtained as a solid (0.093 g, 25%) starting from compound **16a** (0.30 g) and iodobenzene (0.13 ml); m.p. 281–283 °C; ^1^H NMR (400 MHz, DMSO-d_6_): *δ* 2.04–2.07 (m, 2H), 2.57 (t, *J*= 6.4 Hz, 2H), 2.93 (t, *J*= 6.0 Hz, 2H), 6.99 (t, *J*= 7.2 Hz, 1H), 7.09 (d, *J*= 7.6 Hz, 2H), 7.19–7.23 (m, 2H), 7.70 (d, *J*= 8.8 Hz, 2H), 7.94 (d, *J*= 8.8 Hz, 2H), 8.84 (s, 1H), 10.19 (s, 1H), 10.67 (s, 1H). ^13^C NMR (100 MHz, DMSO-d_6_): *δ* 21.0, 31.8, 38.0, 119.3 (2C), 119.5 (2C), 120.2, 124.4, 128.2 (2C), 129.6 (2C), 132.7, 138.1, 143.8, 158.2, 160.4, 174.2, 195.8.

#### N-(4-methoxyphenyl)-4-((5-oxo-5,6,7,8-tetrahydroquinazolin-2-yl)amino)benzenesulfonamide (2)

Compound **2** was obtained as a solid (0.080 g, 20%) starting from compound **16a** (0.30 g) and 1-iodo-4-methoxybenzene (0.26 g); m.p. 233–235 °C. ^1^H NMR (400 MHz, DMSO-d_6_): *δ* 2.05–2.08 (m, 2H), 2.58 (t, *J*= 6.2 Hz, 2H), 2.94 (t, *J*= 6.2 Hz, 2H), 3.66 (s, 3H), 6.79 (d, *J*= 9.2 Hz, 2H), 6.98 (d, *J*= 9.2 Hz, 2H), 7.62 (d, *J*= 8.8 Hz, 2H), 7.93 (d, *J*= 9.2 Hz, 2H), 8.85 (s, 1H), 9.81 (s, 1H), 10.67 (s, 1H). ^13^C NMR (100 MHz, DMSO-d_6_): *δ* 20.8, 31.6, 37.8, 55.3, 114.4 (2C), 115.1, 119.0, 119.1 (2C), 123.3 (2C), 126.1, 127.9 (2C), 130.5, 132.6, 143.4, 156.5, 157.8, 160.5, 195.3.

#### N-(4-nitrophenyl)-4-((5-oxo-5,6,7,8-tetrahydroquinazolin-2-yl)amino)benzenesulfonamide (3)

Compound **3** was obtained as a solid (0.10 g, 25%) starting from compound **16a** (0.30 g) and 1-iodo-4-nitrobenzene (0.28 g); m.p. 264–266 °C. ^1^H NMR (400 MHz, DMSO-d_6_): *δ* 2.05–2.08 (m, 2H), 2.58 (t, *J*= 6.4 Hz, 2H), 2.93 (t, *J*= 6.2 Hz, 2H), 7.30 (d, *J*= 9.2 Hz, 2H), 7.83 (d, *J*= 8.8 Hz, 2H), 7.99–8.02 (m, 2H), 8.13 (d, *J*= 9.2 Hz, 2H), 8.85 (s, 1H), 10.74 (s, 1H), 11.20 (s, 1H). ^13^C NMR (100 MHz, DMSO-d_6_): *δ* 21.0, 31.8, 38.0, 118.2 (2C), 119.4, 119.7 (2C), 125.8 (2C), 128.4 (2C), 131.9, 142.8, 144.4, 144.7, 158.1, 160.4, 174.2, 180.3, 195.9.

#### 4-((5-Oxo-5,6,7,8-tetrahydroquinazolin-2-yl)amino)-N-(4-tolyl)benzenesulfonamide (4)

Compound **4** was obtained as a solid (0.077 g, 20%) starting from compound **16a** (0.30 g) and 1-iodo-4-methylbenzene (0.25 g); m.p. 272–274 °C. ^1^H NMR (400 MHz, DMSO-d_6_): *δ* 2.05–2.08 (m, 2H), 2.17 (s, 3H), 2.58 (t, *J*= 6.8 Hz, 2H), 2.93 (t, *J*= 6.0 Hz, 2H), 6.96–7.03 (m, 4H), 7.66 (d, *J*= 9.2 Hz, 2H), 7.93 (d, *J*= 9.2 Hz, 2H), 8.85 (s, 1H), 10.01 (s, 1H), 10.67 (s, 1H). ^13^C NMR (100 MHz, DMSO-d_6_): *δ* 20.6, 21.0, 31.8, 38.0, 119.2, 119.5 (2C), 120.8 (2C), 128.1 (2C), 129.9 (2C), 132.8, 133.7, 135.4, 143.6, 158.1, 160.4, 174.2, 195.9.

#### 4-((5-Oxo-7-phenyl-5,6,7,8-tetrahydroquinazolin-2-yl)amino)-N-phenylbenzenesulfonamide (5)

Compound **5** was obtained as a solid (0.13 g, 30%) starting from compound **16b** (0.37 g) and iodobenzene (0.23 ml); m.p. 111–113 °C. ^1^H NMR (400 MHz, DMSO-d_6_): *δ* 2.69–2.73 (m, 2H), 2.97–3.10 (m, 2H), 3.52–3.59 (m, 1H), 6.99 (t, *J*= 7.2 Hz, 1H), 7.09 (d, *J*= 8.0 Hz, 2H), 7.19–7.28 (m, 3H), 7.34–7.40 (m, 4H), 7.69 (d, *J*= 8.8 Hz, 2H), 7.96 (d, *J*= 8.8 Hz, 2H), 8.90 (s, 1H), 10.19 (s, 1H), 10.75 (s, 1H). ^13^C NMR (100 MHz, DMSO-d_6_): *δ* 38.3, 44.8, 118.6, 119.3, 119.9 (2C), 124.0, 127.0, 127.1 (2C), 127.9, 128.8 (2C), 129.3 (2C), 132.6, 138.0, 143.3, 143.5, 157.8, 160.5, 172.9, 194.7.

#### N-(4-methoxyphenyl)-4-((5-oxo-7-phenyl-5,6,7,8-tetrahydroquinazolin-2-yl)amino)benzenesulfonamide (6)

Compound **6** was obtained as a solid (0.094 g, 20%) starting from compound **16b** (0.37 g) and 1-iodo-4-methoxybenzene (0.26 g); m.p. 103–105 °C. ^1^H NMR (400 MHz, DMSO-d_6_): *δ* 2.67–2.73 (m, 2H), 2.97–3.01 (m, 2H), 3.53–3.57 (m, 1H), 3.65 (s, 3H), 6.79 (d, *J*= 9.2 Hz, 2H), 6.97 (d, *J* = 9.2 Hz, 2H), 7.26–7.28 (m, 1H), 7.34–7.40 (m, 4H), 7.61 (d, *J*= 8.8 Hz, 2H), 7.95 (d, *J*= 8.8 Hz, 2H), 8.91 (s, 1H), 9.82 (s, 1H), 10.75 (s, 1H). ^13^C NMR (100 MHz, DMSO-d_6_): *δ* 38.4, 44.8, 55.2, 114.3 (2C), 116.8, 118.4, 119.1, 123.2 (2C), 127.0, 127.8 (2C), 128.7, 130.6 (2C), 132.6, 143.2, 156.4, 158.0, 160.5, 194.5.

#### N-(4-nitrophenyl)-4-((5-oxo-7-phenyl-5,6,7,8-tetrahydroquinazolin-2-yl)amino)benzenesulfonamide (7)

Compound **7** was obtained as a solid (0.14 g, 30%) starting from compound **16b** (0.37 g) and 1-iodo-4-nitrobenzene (0.28 g); m.p. 118–120 °C. ^1^H NMR (400 MHz, DMSO-d_6_): *δ* 2.67–2.73 (m, 2H), 2.97–3.04 (m, 2H), 3.54–3.56 (m, 1H), 7.26–7.30 (m, 2H), 7.34–7.40 (m, 3H), 7.82 (d, *J*= 8.8 Hz, 2H), 7.96–8.07 (m, 4H), 8.12 (d, *J*= 9.2 Hz, 2H), 8.91 (s, 1H), 10.82 (s, 1H), 11.21 (s, 1H). ^13^C NMR (100 MHz, DMSO-d_6_): *δ* 38.3, 44.9, 104.5, 118.0 (2C), 118.7, 119.6 (2C), 125.2 (2C), 125.6 (2C), 127.0, 127.1 (2C), 128.2, 128.9 (2C), 139.0, 143.3, 144.0, 157.9, 160.6, 173.0, 194.8.

#### 4-((5-Oxo-7-phenyl-5,6,7,8-tetrahydroquinazolin-2-yl)amino)-N-(4-tolyl)benzenesulfonamide (8)

Compound **8** was obtained as a solid (91 mg, 20%) starting from compound **16b** (0.37 g) and 1-iodo-4-methylbenzene (0.25 g); m.p. 83–85 °C. ^1^H NMR (400 MHz, DMSO-d_6_): *δ* 2.10 (s, 3H), 2.67–2.73 (m, 2H), 2.97–3.04 (m, 2H), 3.54–3.56 (m, 1H), 7.26–7.30 (m, 2H), 7.34–7.40 (m, 3H), 7.82 (d, *J*= 9.2 Hz, 2H), 7.96–8.07 (m, 4H), 8.12 (d, *J*= 9.2 Hz, 2H), 8.91 (s, 1H), 10.82 (s, 1H), 11.21 (s, 1H). ^13^C NMR (100 MHz, DMSO-d_6_): *δ* 20.9, 38.5, 41.7, 45.0, 118.1, 119.4 (2C), 123.1 (2C), 126.4 (2C), 127.2, 127.3 (2C), 129.0 (2C), 130.7 (2C), 132.0, 138.2, 139.8, 142.7, 143.5, 158.1, 161.0, 173.1, 195.1.

#### 4-((7,7-Dimethyl-5-oxo-5,6,7,8-tetrahydroquinazolin-2-yl)amino)-N-phenylbenzenesulfonamide (9)

Compound **9** was obtained as a solid (0.12 g, 30%) starting from compound **16c** (0.32 g) and iodobenzene (0.23 ml); m.p. 215–217 °C. ^1^H NMR (400 MHz, DMSO-d_6_): *δ* 1.03 (s, 6H), 2.46 (s, 2H), 2.86 (s, 2H), 7.00 (t, *J*= 7.2 Hz, 1H), 7.09 (d, *J*= 8.8 Hz, 2H), 7.21–7.24 (m, 2H), 7.70 (d, *J*= 8.8 Hz, 2H), 7.95 (d, *J*= 8.8 Hz, 2H), 8.84 (s, 1H), 10.17 (s, 1H), 10.68 (s, 1H). ^13^C NMR (100 MHz, DMSO-d_6_): *δ* 27.9, 32.4, 44.9, 51.2, 118.0, 119.2 (2C), 120.0, 124.0 (2C), 126.1, 127.9 (2C), 129.2 (2C), 129.8, 132.5, 143.5, 157.4, 160.7, 195.2.

#### 4-((7,7-Dimethyl-5-oxo-5,6,7,8-tetrahydroquinazolin-2-yl)amino)-N-(4-methoxyphenyl)benzenesulfonamide (10)

Compound **10** was obtained as a solid (0.15 g, 35%) starting from compound **16c** (0.32 g) and 1-iodo-4-methoxybenzene (0.26 g); m.p. 210–212 °C. ^1^H NMR (400 MHz, DMSO-d_6_): *δ* 1.03 (s, 6H), 2.86 (s, 2H), 3.65 (s, 3H), 6.79 (d, *J*= 8.8 Hz, 2H), 6.98 (d, *J*= 8.8 Hz, 2H), 7.62 (d, *J*= 8.8 Hz, 2H), 7.93 (d, *J*= 8.8 Hz, 2H), 8.84 (s, 1H), 9.80 (s, 1H), 10.67 (s, 1H). ^13^C NMR (100 MHz, DMSO-d_6_): *δ* 27.8, 32.4, 51.2, 55.2, 114.3 (2C), 119.1 (2C), 123.2 (2C), 127.8 (2C), 129.2, 130.5, 138.1, 143.2, 153.0, 153.8, 160.6, 166.4, 195.1.

#### 4-((7,7-Dimethyl-5-oxo-5,6,7,8-tetrahydroquinazolin-2-yl)amino)-N-(4-nitrophenyl)benzenesulfonamide (11)

Compound **11** was obtained as a solid (0.13 g, 30%) starting from compound **16c** (0.32 g) and 1-iodo-4-nitrobenzene (0.28 g); m.p. 264–266 °C. ^1^H NMR (400 MHz, DMSO-d_6_): *δ* 1.04 (s, 6H), 2.87 (s, 2H), 7.32 (d, *J*= 8.8 Hz, 2H), 7.84 (d, *J*= 8.8 Hz, 2H), 8.02 (d, *J*= 9.2 Hz, 2H), 8.14 (d, *J*= 9.2 Hz, 2H), 8.85 (s, 1H), 10.74 (s, 1H), 11.19 (s, 1H). ^13^C NMR (100 MHz, DMSO-d_6_): *δ* 27.7, 32.3, 44.8, 51.0, 117.7 (2C), 117.9, 119.3 (2C), 125.3 (2C), 127.9 (2C), 131.6, 142.3, 144.0, 144.4, 157.2, 160.5, 172.3, 195.0.

#### 4-((7,7-Dimethyl-5-oxo-5,6,7,8-tetrahydroquinazolin-2-yl)amino)-N-(p-tolyl)benzenesulfonamide (12)

Compound **12** was obtained as a solid (0.10 g, 25%) starting from compound **16c** (0.32 g) and 1-iodo-4-methylbenzene (0.25 g); m.p. 246–248 °C. ^1^H NMR (400 MHz, DMSO-d_6_): *δ* 1.03 (s, 6H), 2.17 (s, 3H), 2.86 (s, 2H), 6.96–7.03 (m, 4H), 7.66 (d, *J*= 8.8 Hz, 2H), 7.93 (d, *J*= 8.8 Hz, 2H), 8.84 (s, 1H), 10.01 (s, 1H), 10.67 (s, 1H). ^13^C NMR (100 MHz, DMSO-d_6_): *δ* 20.4, 27.9, 32.4, 45.9, 51.2, 119.0, 119.2 (2C), 120.4 (2C), 126.1, 127.8 (2C), 129.6 (2C), 130.3, 143.4, 143.8, 144.4, 157.2, 157.4, 195.1.

### CA inhibition assays

An Applied Photophysics stopped-flow instrument was used for assaying the CA catalysed CO_2_ hydration activity^28^. Phenol red (at a concentration of 0.2 mM) was used as indicator, working at the absorbance maximum of 557 nm, with 10 mM Hepes (pH 7.5) as buffer, 0.1 M Na_2_SO_4_ (for maintaining constant ionic strength), following the CA-catalysed CO_2_ hydration reaction for a period of 10–100 s. The CO_2_ concentrations ranged from 1.7 to 17 mM for the determination of the kinetic parameters and inhibition constants. For each inhibitor at least six traces of the initial 5–10% of the reaction have been used for determining the initial velocity. The uncatalysed rates were determined in the same manner and subtracted from the total observed rates. Stock solutions of inhibitors (10 mM) were prepared in distilled-deionised water with 10% of DMSO and dilutions up to 0.01 µM were done thereafter with the assay buffer. The inhibitor and enzyme solutions were preincubated together for 15 min (standard assay at room temperature) prior to assay, to allow for the formation of the E-I complex. The inhibition constants were obtained by non-linear least-squares methods using PRISM 3 and the Cheng–Prusoff equation and represent the mean from at least three different determinations. Enzyme concentrations in the assay system were in the range of 5–12 nM.

### Molecular modelling methods

Docking calculations were executed using the latest AutoDockGPU docking software^29^ along with its GUI AutoDockTools (ADT)^30^. The high-resolution X-ray crystal structure of hCA IX having (PDB 5FL4), hCA I (PDB 6EVR), hCA II (PDB 3K34), and hCA IV (PDB 5JN9)^31–33^ was downloaded and superimposed on the structure of hCA IX. Before docking, the co-crystal ligands of 5FL4, 6EVR, 3K34, and 5JN9 were removed. Later, the protein structures were prepared using the Protein Preparation Wizard of the Maestro suite^34^^,^^35^. The Protein Preparation Wizard deletes water molecules, adds both bond orders and hydrogen atoms, and also produces the appropriate protonation states. Compound **3**, (*S*)-**5**, and (*R*)-**5** were constructed using the 2D sketcher tool of Maestro. The ligands were considered in all the possible flip conformations and appropriate protonation states were obtained. The ligand and protein files were then translated into the AD4 specific format (PDBQT) utilising the scripts prepare_ligand4.py and prepare_receptor4.py, respectively, with all the standard settings. Using AutoDock4_Zn_ forcefield protocol, the script zinc_pseudo.py was applied to add the tetrahedral pseudo atoms to the receptor PDBQT file^36^. The grid boxes were centred on the active site of the protein. While creating the grid parameter files (GPF), the zinc-specific non-bonded pairwise potentials were also included. The dimensions of the grid boxes were defined with a set of grids of 60 Å×40 Å×50 Å along with the spacing of 0.375 Å. A total of 100 independent docking simulations were achieved for every possible conformation of compounds **3**, (*S*)-**5**, and (*R*)-**5**. Each docking calculation comprised 20 million energy evaluations utilising the Lamarckian genetic algorithm local search (GALS) method. All the dockings were performed with a population size of 250 and 300 runs of Solis and Wets local search with a probability of 0.6. A rate of mutation of 0.02 and a crossover rate of 0.8 were used to produce new docking attempts for following generations, and the best individual from each generation was propagated over the following generation. The docking modes of every 100 independent docking calculations of the ligand binding to the metalloenzyme were clustered based on a 2 Å cut-off value based on Cartesian coordinates of the atom and were also ranked based on free energy binding (Δ*G*_AD4_). Compounds **3**, (*S*)-**5**, and (*R*)-**5** best conformations were selected based upon the predicted Δ*G*_AD4_ as well the cluster population.

## Results and discussion

### Chemistry

**Scheme 1.**
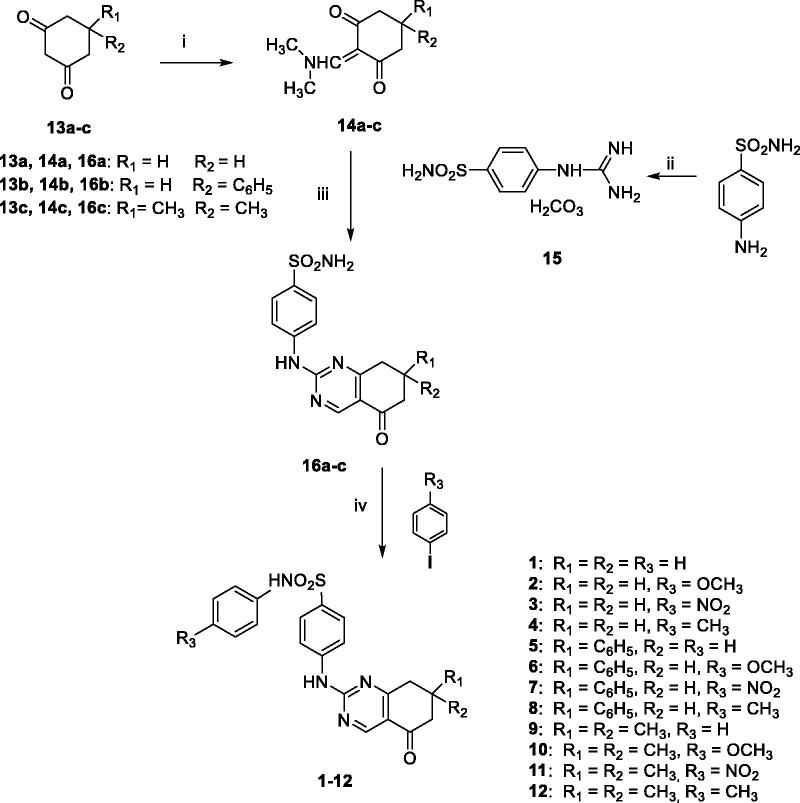
Reagents and conditions: (i) DMF-DMA, 100 °C, 1 h; (ii) conc HCl/50% cyanamide water solution, 100 °C, 0.5 h; (iii) *n*-butanol, NaOH, M.W. (40 min, 160 °C); (iv) MeCN, K_2_CO_3_, DMEDA, CuI, 100 °C, 8–24 h.

The key intermediates for the synthesis of all the new target compounds **1**–**12** were the bicyclic tetrahydroquinazoline derivatives **16a**–**c** already described by us^15^ belonging to class **V** (Chart 1). However, the synthetic procedure to obtain compounds **16a**–**c** has been here improved by exploiting MW irradiation as a valuable alternative to conventional heating, allowing to reach higher yields and shorter reaction times (40 min vs. 16 h). In detail, the commercially available 5-substituted-1,3-cyclohexandiones **13a**–**c** were reacted with an excess of *N*,*N*-dimethylformamide-dimethyl acetal (DMF-DMA) at 100 °C for 1 h, to furnish the intermediates **14a**–**c**. Then, MW-assisted condensation reaction between the proper bielectrophyle 2-[(dimethylamino)methylene]cyclohexan-1,3-dione **14a**–**c**^15^ and the binucleophile guanidine **15**^14^ was performed to obtain crude compounds **16a**–**c** which were purified by recrystallisation from ethanol (Scheme 1).

The quinazolyl-*N*-arylbenzenesulfonamides **1**–**12** were then obtained by reacting compounds **16a**–**c** with the proper *p*-substituted phenyliodide in acetonitrile (MeCN) in the presence of K_2_CO_3_, *N*,*N*′-dimethylethylendiamine (DMEDA), and CuI. The reaction mixture was heated at 100 °C and stirred for 8–24 h (TLC analysis); after cooling, the suspension obtained was filtered under reduced pressure and the solid was purified by flash chromatography, yielding the pure target derivatives **1**–**12**.

### CA inhibition assays and structure–activity relationships

All the newly synthesised compounds **1**–**12** were investigated for their enzyme inhibitory ability against four physiologically relevant CA isoforms, namely the human hCA I, II, IV, and IX (Table 1), by a stopped-flow CO_2_ hydrase assay using acetazolamide (**AAZ**, 5-acetamido-1,3,4-thiadiazole-2-sulphonamide, Chart 1) as the standard drug^28^. Table 1 also reports the data referring to the parent primary sulphonamides **16a**–**c**^15^ for comparison purposes.

**Table 1.** Inhibition of hCA isoforms I, II, IV, and IX with tetrahydroquinazoline derivatives **1**–**12**, **16a**–**c**, and **AAZ** as the reference standard by a stopped-flow CO_2_ hydrase assay.

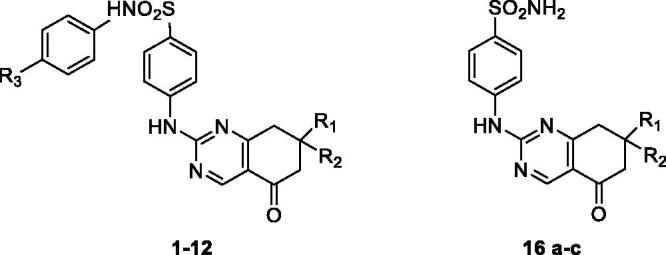							
Compound	R_1_	R_2_	R_3_	*K*_i_ (µM)^a^			
				hCA I [CA I/CA IX]^b^	hCA II [CA II/CA IX]^b^	hCA IV [CA IV/CA IX]^b^	hCA IX
**1**	H	H	H	89.1 [185.6]	41.2 [85.8]	>100 [>208]	0.48
**2**	H	H	OCH_3_	>100 [>217]	50.3 [109.3]	>100 [>217]	0.46
**3**	H	H	NO_2_	>100 [>238]	>100 [> 238]	>100 [>238]	0.42
**4**	H	H	CH_3_	>100 [>96]	>100 [> 96]	>100 [>96]	1.04
**5**	H	C_6_H_5_	H	>100 [>222]	>100 [> 222]	>100 [>222]	0.45
**6**	H	C_6_H_5_	OCH_3_	34.0 [19.6]	11.5 [6.6]	>100 [>57.8]	1.73
**7**	H	C_6_H_5_	NO_2_	>100 [>75.2]	67.5 [50.7]	>100 [>75.2]	1.33
**8**	H	C_6_H_5_	CH_3_	46.1	13.0	>100	15.7
**9**	CH_3_	CH_3_	H	70.5	7.4	91.1	47.3
**10**	CH_3_	CH_3_	OCH_3_	24.7	5.1	78.1	48.1
**11**	CH_3_	CH_3_	NO_2_	62.0	6.9	>100	>100
**12**	CH_3_	CH_3_	CH_3_	84.9	22.4	62.1	19.3
**16a** ^c^	H	H	–	0.0639 [116]	0.0052 [9.45]	0.073 [132.7]	0.00055
**16b** ^c^	H	C_6_H_5_	–	>100 [869]	0.0060 [7.6]	0.078 [98.7]	0.00079
**16c** ^c^	CH_3_	CH_3_	–	>100 [712]	0.0082 [9.2]	0.3194 [359]	0.00089
**AAZ**				0.25	0.012	0.074	0.026

^a^ Mean from three different assays, by a stopped flow technique (errors were in the range of ±5–10% of the reported values).

^b^ *K*_i_ ratio for the indicated enzyme isoforms.

^c^ Data from ref.^15^

In particular, the inhibitory profile of the tetrahydroquinazolin-2-yl-amino-benzenesulfonamides **1**–**12** appears strictly dependent on the substitution pattern at 7-position, with few exceptions. Unsubstituted compounds (**1**–**4**) exhibit high potency against hCA IX (*K*_i_ values ranging from 0.42 μM to 1.04 μM), combined with an excellent selectivity with respect to the other isoforms tested. This trend appears to be independent of the decoration of the pendant phenyl ring (R_3_).

The inhibitory potency of 7-phenyl derivatives (**5**–**8**) appears to be closely related to the decoration on the benzenesulfonamide moiety (R_3_). hCA IX inhibition potency was maintained high when position 1 is unsubstituted (hCA IX, **5** *K*_i_ 0.45 vs. **1** *K*_i_ 0.42), and slightly decreased when R_3_=OCH_3_ or NO_2_ (hCA IX, **6** *K*_i_ 1.73 and **7** *K*_i_ 1.33 vs. **2** *K*_i_ 0.46 and **3** *K*_i_ 0.42, respectively). The hCA IX-selectivity profile varied from excellent to good moving from R_3_=H to R_3_=NO_2_ and R_3_=OCH_3_. Specifically, the best performing compound of the subseries in terms of hCA IX inhibitory potency and selectivity is the unsubstituted (R_3_=H) compound **5**; the *p*-nitrophenyl-substituted analogue **7** (R_3_=NO_2_) results in a quite potent (*K*_i_ 1.33 µM) and selective (*K*_i_ ratio CAI/CAIX >75.2, CAII/CAIX 50.7, CAIV/CAIX >75.2) hCA IX inhibitor; the *p*-methoxy-substituted **6** (R_3_=OCH_3_), retains the hCA IX inhibitory potency of the same order of magnitude of **7** (*K*_i_ 1.73 µM), but the selectivity towards this isoform is considerably reduced (*K*_i_ ratio CAI/CAIX >19.6, CAII/CAIX 6.6, CAIV/CAIX >57.8). The presence of a methyl group at R_3_ produced a marked reduction in hCA IX inhibitory potency (hCA IX: **8** *K*_i_ 15.7 vs. **4** *K*_i_ 1.04), and a loss of selectivity, being **8** equally potent on hCA II and hCA IX.

Insertion of a double methyl substitution at position 7 produced a dramatic drop in hCA IX inhibition potency and a general gain in activity towards the other hCAs tested, particularly for hCA II. The 7,7-dimethyl-quinazolinyl-*N*-arylbenzenesulfonamides **9**–**12** inhibited all the CAs tested with micromolar potency (with few exceptions), showing a moderate selectivity for the hCA II isoform (*K*_i_ values from 5.1 to 22.4 µM). Only compound **12** (R_3_=CH_3_) did not register a gain in selectivity for the hCA II isoform showing comparable *K*_i_ values for all the tested hCA isoforms.

All in all, these data highlighted the identification of several hCA IX inhibitors with high potency and selectivity, featuring a secondary benzenesulfonamide on the tetrahydroquinazoline scaffold. The best-performing compounds are represented by **3** (R_1_=R_2_=H, R_3_=NO_2_) and **5** (R_1_=H, R_2_=C_6_H_5_, R_3_=H), that are the most potent (**3** *K*_i_ 0.42 µM; **5** *K*_i_ 0.45 µM) and selective (*K*_i_ ratio CAI/CAIX, CAII/CAIX, CAIV/CAIX >238.1 and >222.2 for **3** and **5**, respectively) hCA IX inhibitors of this new series of derivatives.

It should be outlined that, comparing the most interesting new compounds **1**–**5** with the parent primary tetrahydroquinazole sulphonamides **16a**–**c**, despite the lower hCA IX inhibitory potency of the secondary **1**–**5**, their hCA IX selectivity is greater than that observed for primary sulphonamides **16a**–**c**. This greater selectivity is especially evident with respect to isoform II with *K*_i_ ratios CAII/CAIX ranging from 7.6 to 9.45 for **16a**–**c** and from 85.8 to >238 for **1**–**5**.

Furthermore, a comparison of the obtained results for **1**–**4** with those of the corresponding tetrahydroindazole-based secondary sulphonamides of series **VI** (**VIa**–**d** in Table 2) recently described by us^16^, highlighted that, by keeping the same R_3_-substituents (R_3_=H, *p*-OCH_3_, *p*-NO_2_, *p*-CH_3_) and changing the scaffold from tetrahydroindazole (**VIa**–**d)** to tetrahydroquinazole (**1**–**4**), the good potency (*K*_i_ 1.4–5.7 µM) and selectivity towards the hCA I isoform shifted to high potency (*K*_i_ 0.42–1.04 µM) and excellent selectivity towards the hCA IX isoform. In particular, by comparing the *p*-nitro-substituted derivatives **3** and **VIc** (R_1_=R_2_=H, R_3_=NO_2_) which are the best performing compounds in the two subseries, a *K*_i_ value of 5.7 µM for **VIc** against hCA I with slight selectivity (*K*_i_ ratio CAII/CAI 8.8, CAIV/CAI 13.3, CAIX/CAI 14.7), and a *K*_i_ value of 0.42 µM against the hCA IX isoform with a high selectivity (*K*_i_ ratio CAI/CAIX, CAII/CAIX, CAIV/CAIX >238.1) for **3** are highlighted.

**Table 2.** Inhibition of hCA isoforms I, II, IV, and IX with tetrahydroquinazoline (**1**–**4**) tetrahydroindazole (**VIa**–**d**)^16^ and secondary sulphonamides.

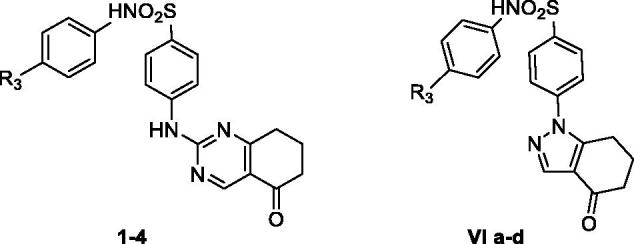					
Compound	**R_3_**	*K*_i_ (µM)^a^			
		hCA I	hCA II	hCA IV	hCA IX
**1**	H	89.1 [CA I/CA IX]^b^=185.6	41.2 [CA II/CA IX]^b^=85.8	>100 [CA IV/CA IX]^b^>208	0.48
**2**	OCH_3_	>100 [CA I/CA IX]^b^>217	50.3 [CA II/CA IX]^b^=109.3	>100 [CA IV/CA IX]^b^>217	0.46
**3**	NO_2_	>100 [CA I/CA IX]^b^>238	>100 [CA II/CA IX]^b^>238	>100 [CA IV/CA IX]^b^>238	0.42
**4**	CH_3_	>100 [CA I/CA IX]^b^>96	>100 [CA II/CA IX]^b^>96	>100 [CA IV/CA IX]^b^>96	1.04
**VIa** ^c^	H	5.7	31.0 [CA II/CA I]^b^=5.4	59.6 [CA IV/CA I]^b^=10.4	97.9 [CA IX/CA I]^b^=17.2
**VIb** ^c^	OCH_3_	1.4	5.9 [CA II/CA I]^b^=4.2	79.3 [CA IV/CA I]^b^=56.6	56.5 [CA IX/CA I]^b^=40.3
**VIc** ^c^	NO_2_	5.7	50.3 [CA II/CA I]^b^=8.8	75.8 [CA IV/CA I]^b^=13.3	83.7 [CA IX/CA I]^b^=14.7
**VId** ^c^	CH_3_	4.9	8.4 [CA II/CA I]^b^=1.71	52.5 [CA IV/CA I]^b^=10.7	39.2 [CA IX/CA I]^b^=8.0
**AAZ**		0.25	0.012	0.074	0.026

a Mean from three different assays, by a stopped flow technique (errors were in the range of ±5–10% of the reported values).

b *K*_i_ ratio for the indicated enzyme isoforms.

c Data from ref.^16^

### Molecular modelling studies

**Figure 1.**
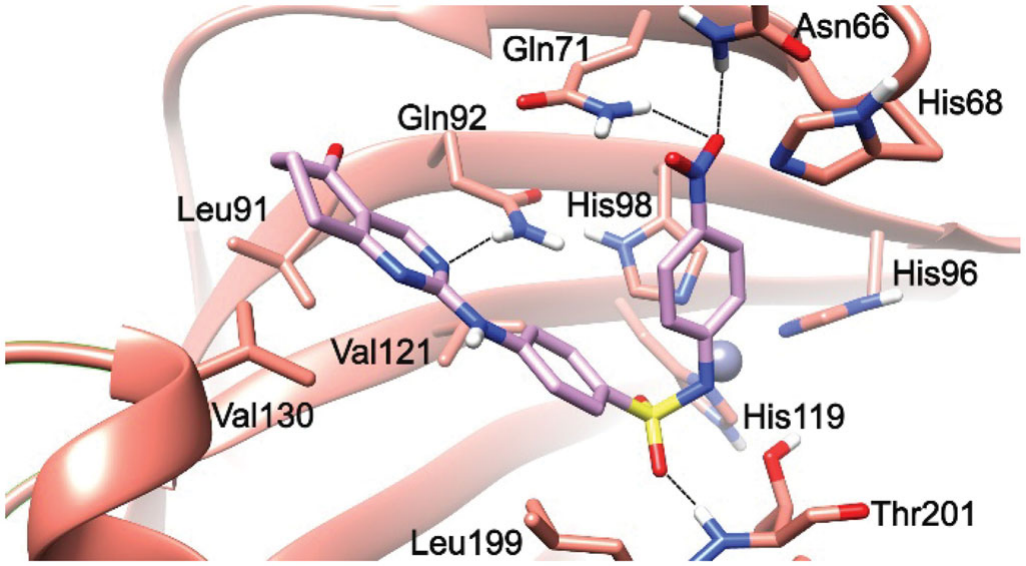
**3**/hCA IX theoretical complex (PDB 5FL4) computed by docking calculations. The protein is shaded in salmon and all the critical residues are labelled. The ligand is shown in pink colour while the H-bonds are shown in black dashed lines.

To rationalise the SAR data attained for various substitutions of newly designed secondary sulphonamides (Table 1), a series of molecular modelling studies were performed on compounds **3** and **5**, as they are the most potent inhibitors of hCA IX as well as the most selective over the other tested hCAs (I, II, and IV) of the whole set. In this process, the AutoDock4_Zn_ docking protocol, devised for docking experiments on zinc-chelating ligands, was employed as implemented in our previous reports on hCAIs^15^^,^^37^. The best docking solution, i.e. the one having the lowest predicted binding free energy, achieved for compound **3** in the hCA IX **(**PDB 5FL4)^31^ binding site is shown in Figure 1.

Mainly, the sulphonamide chelates the catalytic zinc ion through its negatively charged nitrogen atom in a geometry that is consistent with the other CAIs. Additionally, one of the sulphonamide oxygen atoms is well-positioned to accept an H-bond from the backbone NH of Thr201. Apart from these interactions, which were consistent with our previous reports on hCAs^15^^,^^37^, it is also interesting to see that the phenyl ring, attached to the sulphonamide negative nitrogen, forms a π–π interaction with His98 and places its *p*-NO_2_ group in a hydrophilic protein region at H-bonding distance to multiple residues such as Asn66, His68, and Gln71. These latter interactions might explain why compound **4**, featuring the lipophilic *p*-CH_3_ group, by establishing less favourable interactions with the enzyme counterpart, is less active than compounds **1**–**3**. Interestingly, the same trend was recorded for the phenyl-substituted analogues **5**–**8**. The benzene ring of the benzenesulfonamide forms van der Waals interactions with Val121 and Leu199 while the attached pyrimidine ring of the tetrahydroquinazoline scaffold forms an H-bond with Gln92 residue. This latter interaction might explain why the tetrahydroquinazolines described in the present work are generally more potent than the previously reported tetrahydroindazoles^16^. Moreover, the oxygen atom enclosed in tetrahydroquinazoline is pointing towards the “selectivity hotspot” of the hCA IX binding site.

**Figure 2.**
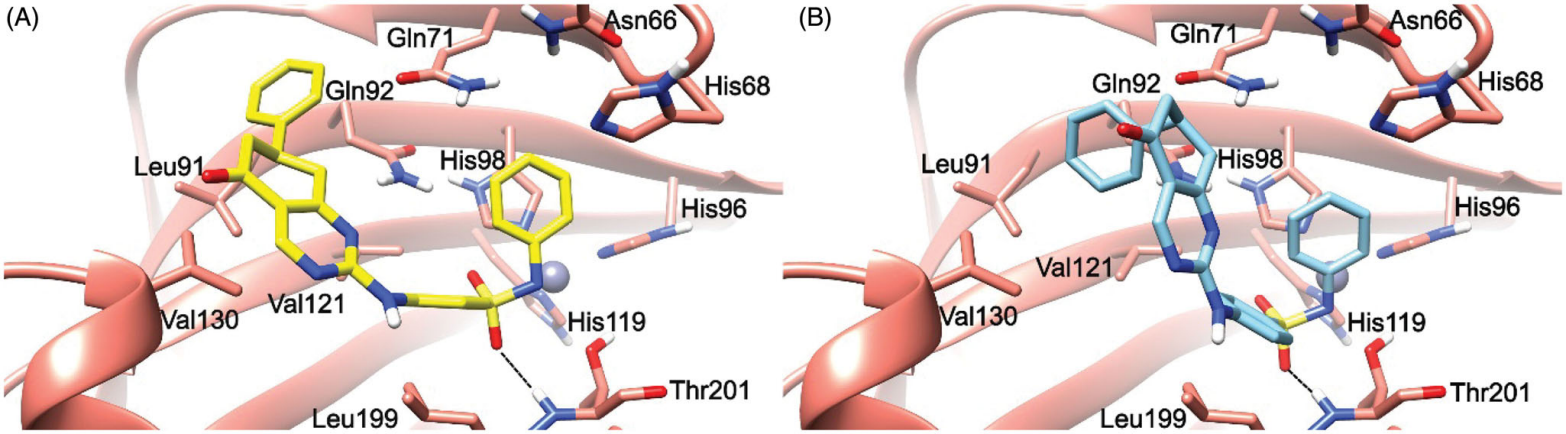
(*S*)-**5**/hCA IX (A) and (*R*)-**5**/hCA IX theoretical complexes (PDB 5FL4) computed by docking calculations. The protein is shaded in salmon and all the critical residues are labelled. The ligand is shown in yellow and cyan, respectively, while the H-bonds are shown in black dashed lines.

Dockings of both isomers of **5** allow postulating the absence of a stereoselective recognition by hCAIX as, in both cases, the predicted lowest energy conformation places the negatively charged sulphonamide nitrogen to chelate the Zn^2+^ (Figure 2) with an overall orientation resembling the one achieved for **3**.

In particular, the (*S*)-**5** docking suggests that the negative nitrogen connected to the secondary sulphonamide is chelating the zinc ion, while a sulphonamide oxygen is forming an H-bond with the backbone NH of Thr201. If compared to the binding pose achieved for **3**, the presence of the phenyl ring at position 7 of the tetrahydroquinazoline ring seems to induce a partial relocation of the ligand inducing the loss of the H-bond interaction with Gln92, thereby explaining why compounds **5**–**8** are less active than their unsubstituted counterparts **1**–**4**. In (*S*)-**5**, this loss seems to be counterbalanced by the presence of several van der Waals contacts between the phenyl ring and Leu91, Gln92, and Val130 enzyme residues in the “selectivity hotspot”. (*R*)-**5** docking calculations indicate the presence of a similar interaction pattern (Figure 2(B)).

**Figure 3.**
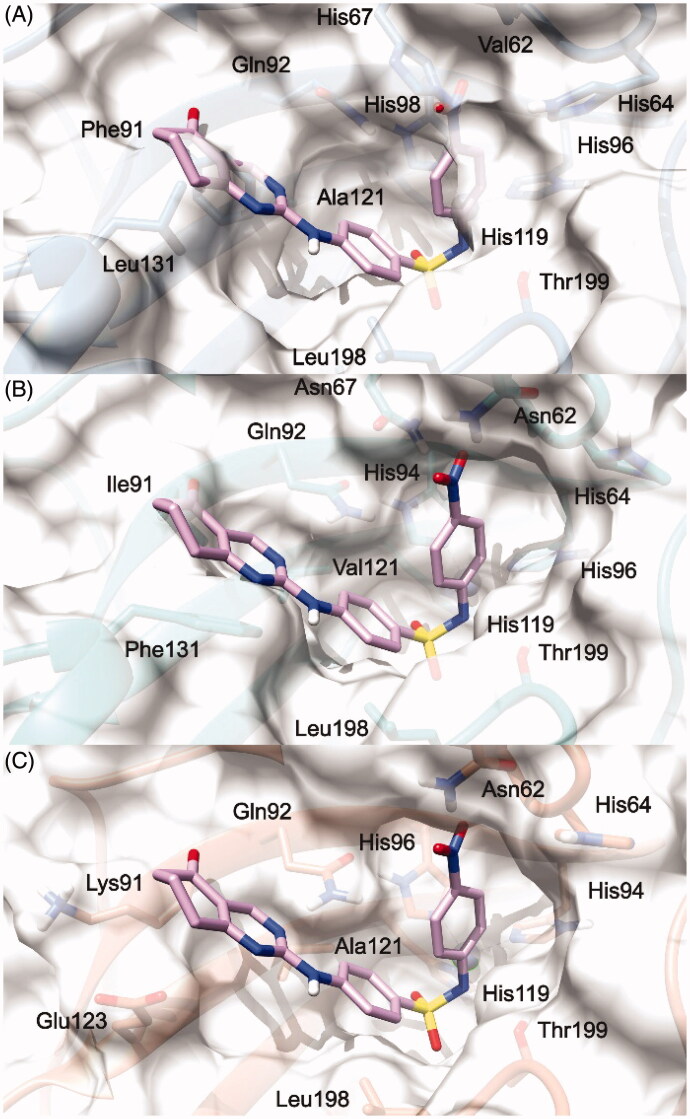
**3**/hCA IX theoretical binding pose translated into hCA I (PDB 6EVR, A), hCA II (PDB 3K34, B), and hCA IV (PDB 5JN9, C) structures. The proteins are shown in blue, sea-green, and coral ribbons and sticks, respectively, with their molecular surface shaded in white. The ligand is depicted in pink.

**Figure 4.**
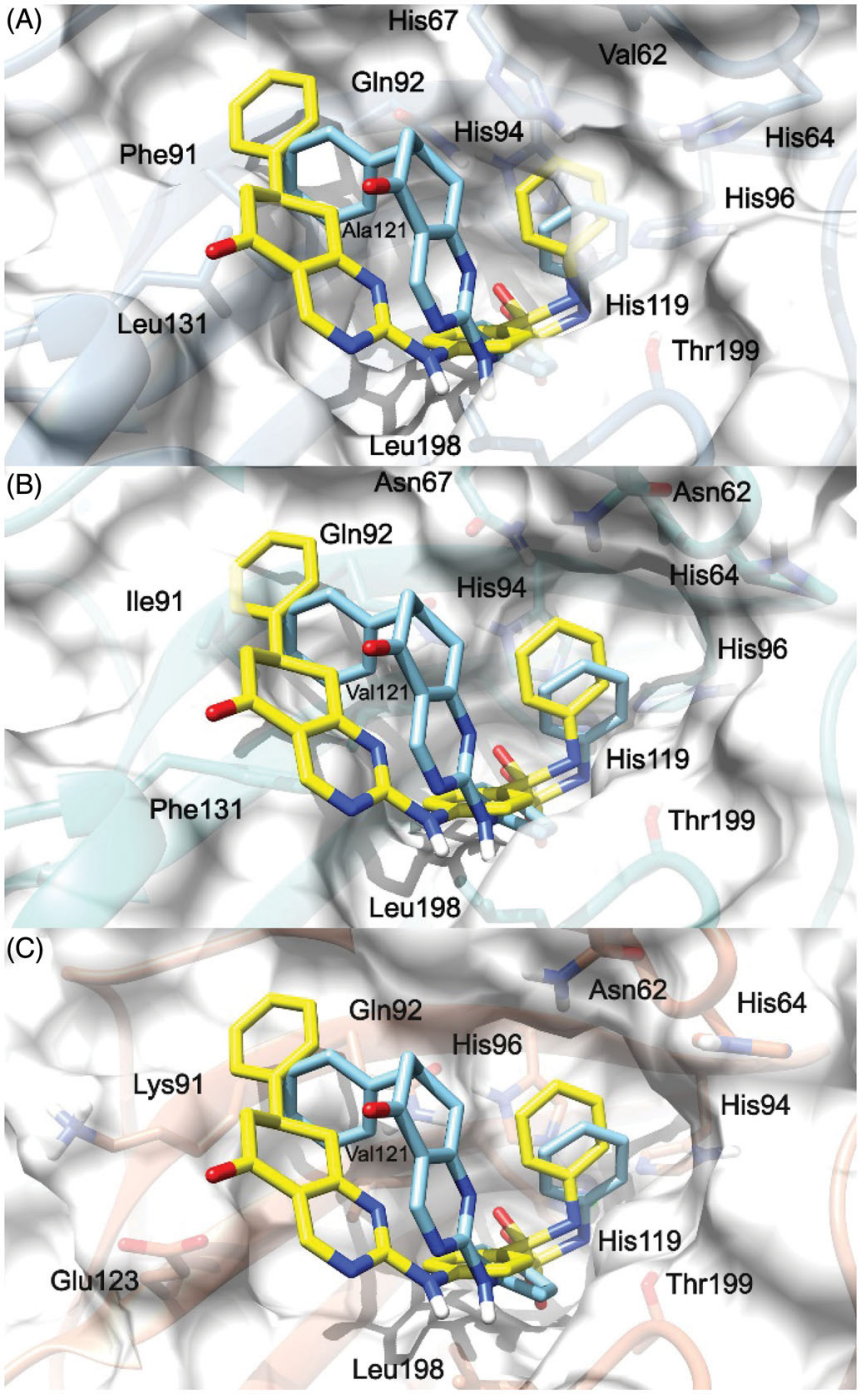
(*S*)-**5** and (*R*)-**5**/hCA IX theoretical binding pose translated into hCA I (PDB 6EVR, A), hCA II (PDB 3K34, B), and hCA IV (PDB 5JN9, C) structures. The proteins are shown in blue, sea-green, and coral ribbons and sticks, respectively, with their molecular surface shaded in white. (*S*)-**5** and (*R*)-**5** are depicted in yellow and cyan sticks. All the pictures were rendered using UCSF chimera molecular visualisation software^40^.

To explain the selectivity profiles of **3**, (*S*)-**5**, and (*R*)-**5**, docking calculations of these compounds were also attempted employing the published structures of hCA I (PDB 6EVR), hCA II (PDB 3K34), and hCA IV (PDB 5JN9)^32^^,^^33^. Interestingly, analysis of the obtained results revealed a certain difficulty in predicting a binding pose featuring the chelation of the Zn^2+^ ion. To explain these theoretical results, the binding pose obtained for **3**, (*S*)-**5**, and (*R*)-**5** into the hCA IX structure and conducive of the Zn^2+^ chelation, was rigidly translated into the aligned binding sites of hCA I, hCA II, and hCA IV. From this analysis, it is clear that such a ligand orientation is unfeasible into the hCA I, hCA II as it would give rise to multiple steric clashes (Figures 3(A,B) and 4(A,B)). Concerning hCA IV, no steric clashes were evidenced, while unfavourable electrostatic interactions between the hydrophilic “selectivity hotspot” of hCA IV and the ligands could be detected (Figures 3(C) and 4(D)). This should explain while compounds **3** and **5** are unable to efficiently inhibit hCA I, hCA II, and hCA IV.

## Conclusions

Within our project aimed at investigating the chemical space of secondary sulphonamide CAIs, we herein reported the design, synthesis, and biological evaluation of a focussed library of variously decorated *N*-phenyl secondary sulphonamides featuring the bicyclic tetrahydroquinazole scaffold. Exploiting SAR studies from our previous work, we identified several compounds showing submicromolar potency and excellent selectivity for hCA IX, an isoform that has attracted significant interest as a putative cancer therapeutic target. SAR studies highlighted a strict correlation between the activity/selectivity profile and the substitution patterns at the 7-position of the central scaffold and the para-position of the benzenesulfonamide moiety of the target derivatives. Molecular modelling studies rationalised the SARs in terms of both inhibitory activity and selectivity profile. Overall, this study provided further information on the structural requirements for efficient and selective inhibition of the cancer-related hCA IX isoform and it could be useful for the design of novel anticancer agents. The class of secondary sulphonamides, although less investigated for their CA inhibitory properties, may lead to relevant medicinal chemistry discoveries. Moreover, very recently Kennedy et al.^38^ investigated inflammasome and pyroptotic pathways and identified a novel photoaffinity alkyne-tagged probe for such processes, compound belonging to the secondary sulphonamide class, which is also targeting efficiently CA II, confirming thus our earlier data^16^^,^^25^^,^^39^ that secondary sulphonamides effectively inhibit these enzymes, by a mechanism which starts to be elucidated through this and the above-mentioned works.
